# High-Density Exploration of Activity States in a Multi-Area Brain Model

**DOI:** 10.1007/s12021-023-09647-1

**Published:** 2023-11-20

**Authors:** David Aquilué-Llorens, Jennifer S. Goldman, Alain Destexhe

**Affiliations:** 1 Paris-Saclay University, CNRS, Paris-Saclay Institute of Neuroscience (NeuroPSI), 91400 Saclay, France; 2 Starlab Barcelona SL, Neuroscience BU, Av Tibidabo 47 bis, Barcelona, Spain

**Keywords:** Cerebral cortex, Computational models, Asynchronous states, Information processing, Whole-brain model, Parameter exploration, Brain states, High-performance computing

## Abstract

To simulate whole brain dynamics with only a few equations, biophysical, mesoscopic models of local neuron populations can be connected using empirical tractography data. The development of mesoscopic mean-field models of neural populations, in particular, the Adaptive Exponential (AdEx mean-field model), has successfully summarized neuron-scale phenomena leading to the emergence of global brain dynamics associated with conscious (asynchronous and rapid dynamics) and unconscious (synchronized slow-waves, with Up-and-Down state dynamics) brain states, based on biophysical mechanisms operating at cellular scales (e.g. neuromodulatory regulation of spike-frequency adaptation during sleep-wake cycles or anesthetics). Using the Virtual Brain (TVB) environment to connect mean-field AdEx models, we have previously simulated the general properties of brain states, playing on spike-frequency adaptation, but have not yet performed detailed analyses of other parameters possibly also regulating transitions in brain-scale dynamics between different brain states. We performed a dense grid parameter exploration of the TVB-AdEx model, making use of High Performance Computing. We report a remarkable robustness of the effect of adaptation to induce synchronized slow-wave activity. Moreover, the occurrence of slow waves is often paralleled with a closer relation between functional and structural connectivity. We find that hyperpolarization can also generate unconscious-like synchronized Up and Down states, which may be a mechanism underlying the action of anesthetics. We conclude that the TVB-AdEx model reveals large-scale properties identified experimentally in sleep and anesthesia.

**Supplementary Information:**

The online version contains supplementary material available at 10.1007/s12021-023-09647-1.

## Introduction

Consciousness is fundamental to human existence: it is experiencing, having a thought, being aware. Unconsciousness is not unfamiliar either: naturally, we lose consciousness when going into dreamless (NREM) sleep, or medically, through external factors like general anesthesia for surgery or brain injury. Furthermore, disorders of consciousness, such as unresponsive wakefulness syndrome or coma, as well as disorders of the wake-sleep cycle, such as insomnia, take a great toll on society (Evers, 2016; Wade, 2010). Moreover, approximately 200 million surgical procedures are carried out under anesthesia each year (Weiser et al., 2008). However, understanding from which multi-scale mechanisms consciousness emerges remains an active field of philosophical and scientific inquiry (Koch et al., 2016; Northoff & Lamme, 2020; Wu, 2018).

Although no particular brain region or mechanism can uniquely account for states of consciousness (Koch et al., 2016), it is possible to roughly categorize the macroscopic activity of the brain into conscious-like (during dreaming or being awake) and unconscious-like (during deep sleep and under general anesthesia) states (Goldman et al., 2019).

Macroscopic recordings of electromagnetic brain activity (such as EEG and MEG) during conscious states typically show low amplitude, complex, disorganized signals occurring at relatively high frequencies (Niedermeyer & Lopes Da Silva, 2004). Conversely, unconscious states exhibit higher amplitude, simpler, synchronous, oscillatory, low frequency activity (Goldman et al., 2019; Steriade et al., 1993).

Interactions between brain areas are strongly similar to anatomical white matter tracts during unconscious states, while, in conscious states, more complex brain activity explores a richer repertoire of functional configurations that are less constrained by global brain structure (Barttfeld et al., 2015; Tagliazucchi et al., 2015). The complex and disorganized activity of conscious states renders the brain more globally responsive to external stimuli (Destexhe et al., June 1999; Massimini et al., 2005). In contrast, during unconscious states, responses to stimuli remain localized, with more restricted propagation between non-adjacent cortical regions (Massimini et al., 2005).

There are also observable differences between conscious and unconscious states at the neuronal scale. During consciousness, cortical neurons exhibit sustained, but sparse and irregular firing patterns characterized as Asynchronous Irregular (AI) activity. In contrast, during periods of unconsciousness, cortical neurons more synchronously oscillate between hyperpolarized (Down) states and depolarized and AI-like firing (Up) states (Goldman et al., 2019; Steriade et al., 1993; Destexhe, 2009).

Differences in neuron behavior between brain states can result from changes in ambient neuromodulator concentrations that regulate neuronal spike-frequency adaptation, resulting in more or less synchronous activity with different characteristic frequency distributions (McCormick, 1992; Steriade & McCarley, 2005). Spike-frequency adaptation is a self-inhibiting process in which neurons decrease their firing rate in response to sustained activity, sometimes to the point of silencing the neuron (Gutkin & Zeldenrust, 2014). During conscious states, concentrations of neuromodulators such as acetylcholine are higher (McCormick, 1992), effectively suppressing spike frequency adaptation and facilitating AI firing patterns. During unconscious states, such neuromodulator concentrations are lower, increasing spike frequency adaptation, thus entraining neurons into alternating periods of silence and activity (McCormick, 1992; Steriade & McCarley, 2005).

Understanding the relationships between macroscopic global dynamics and microscopic neuromodulatory processes and neuronal network activity, both in conscious and unconscious brain states, can help the scientific community shed light onto the puzzling concept of consciousness. This is the principal goal of the present study, to explore the relation between microscopic parameters with the emergence of properties at large-scales in the brain.

An essential tool to realize this exploration, is the TVB-AdEx model, introduced by Goldman and colleagues, which consists of a multi-scale whole brain model that connects the behavior of individual neurons to whole brain dynamics (Goldman et al., 2020, 2023). To build this model, one starts at the microscopic scale by simulating a spiking network containing excitatory and inhibitory Adaptive Exponential (AdEx) integrate and fire neuron models (Brette & Gerstner, 2005). Then, the mesoscopic AdEx mean-field model is derived from this network, which captures the aggregated spiking dynamics of the network at mesoscopic scales (El Boustani & Destexhe, 2009; Zerlaut et al., 2017; di Volo et al., 2019). In order to bridge mesoscopic to macroscopic scales, networks of neurons, represented as AdEx mean-field models, are connected according to anatomical tractography data, with model delays informed by the properties of the tracts. The TVB-Adex is therefore a multi-scale model capable of exhibiting clear transitions in dynamical states, from fast-oscillating low-amplitude complex (AI) states to slow-oscillating high-amplitude Up and Down (UD) states, when changing the adaptation value that simulates changes in acetylcholine concentration (Destexhe, 2009). The model is also capable of reproducing different evoked patterns of activity during simulated conscious and unconscious states (Goldman et al., 2020, 2023), consistent with empirical experiments (Destexhe et al., 1999; Massimini et al., 2005; Casali et al., 2013).

The TVB-AdEx model is a high-dimensional dynamical model with a multitude of parameters that should be understood, estimated, and the resulting dynamical behavior characterized. Fortunately, many parameters can be informed through physiological and mathematical constraints (El Boustani & Destexhe, 2009; di Volo et al., 2019; Zerlaut et al., 2017). However, the values of at least five parameters can still vary within a considerable range, allowing the model to explore many, possibly interesting, regimes. Thoroughly studying the effects and interactions of five different parameters within such a complex network presents a computationally-costly challenge that had not yet been addressed. Such a thorough exploration is necessary to explore the emerging properties of the system at large-scales, which is our aim here.

In this paper, we provide such an exploration, using a detailed parameter scan realized on High Performance Computing (HPC) systems. We show the different states (including pathological states) that emerge at large scales, depending on the model’s microscopic parameters. We also characterize properties of brain-scale dynamics, reporting changes in functional connectivity and its relation to structural connectivity resembling global neural dynamical properties of experimental measurements of the brain in varying states of consciousness. In sum, we report the exploration and openly offer code to run parallel simulations of global human brain dynamics using the parallelized TVB-AdEx model with HPC.

## Methods

### The TVB-AdEx Model

The TVB-AdEx model is a biologically informed, brain-scale cortical model built using The Virtual Brain (TVB) platform. Using MRI scans, one can divide a scanned brain into different anatomical regions and estimate the strength of the connections between regions using tractography analyses (Schirner et al., 2015). From this data one can build the TVB-AdEx model as a network of AdEx mean-field models, each mean-field model describing the neuronal activity of one of the anatomical regions in the scanned brain. The strength of the interactions between the mean-field models are determined by the connectivity matrix, also called connectome, obtained from the MRI tractography analyses. The parcellation used for this model divides the brain into 68 regions (berlinSubjects/QL_20120814 from https://zenodo.org/record/4263723, Schirner et al. (2015)) and, therefore, 68 AdEx mean-field models make up the TVB-AdEx model.

Each one of the 68 AdEx mean-field models reproduces the mean behavior of a spiking network made up of $10^4$ Adaptive Exponential (AdEx) integrate and fire neurons (80% of them being excitatory and the other 20% inhibitory) (di Volo et al., 2019). Through a Master equation formalism (El Boustani & Destexhe, 2009), the mean-field model describes the general activity of the neural populations in the spiking network with seven differential equations (di Volo et al., 2019; Zerlaut et al., 2017). When spike-frequency adaptation is low (simulating conscious brain states with high levels of neuromodulation (di Volo et al., 2019)) the mean-field model is able to reproduce AI states through noisy perturbations around a stable non-zero fixed point (Up fixed point). When adaptation is increased (simulating reduced neuromodulation in the brain), the model also reproduces Up-Down states by cyclically traveling between the fixed point at the origin (Down fixed point) and the Up fixed point (di Volo et al., 2019).

Note that there are two different networks needed to build this macroscopic model: the spiking network, modeling $10^4$ neurons at the microscale, and the TVB-AdEx network, consisting of the 68 inter-connected AdEx mean-field models. From now on, we will only refer to the spiking network when the term "spiking" is explicitly mentioned, in any other case, the word "network" will refer to the TVB-AdEx model.

The variables that describe the mean activity of the excitatory and inhibitory populations are the mean excitatory and inhibitory firing rate, $\nu _e$ and $\nu _i$, respectively. Under normal working conditions, both mean firing rates remain under 100 Hz, typically in the range [0, 50] Hz. However, the dynamical landscape of the AdEx mean-field model also contains a fixed point at a pathologically high level of activity, around 190 Hz, where neurons in the populations fire immediately after their refractory period (see Fig. 14). This fixed point does not disappear when building the TVB-AdEx network. Instead it becomes more difficult to detect under which conditions one, or multiple, mean-fields in the network stabilize at that pathological point, an event that we will refer to as "paroxysmal firing rates" or "paroxysmal fixed points". We argue later that these paroxysms are more relevant to epileptic states, so they were avoided in this study.

**Fig. 1:**
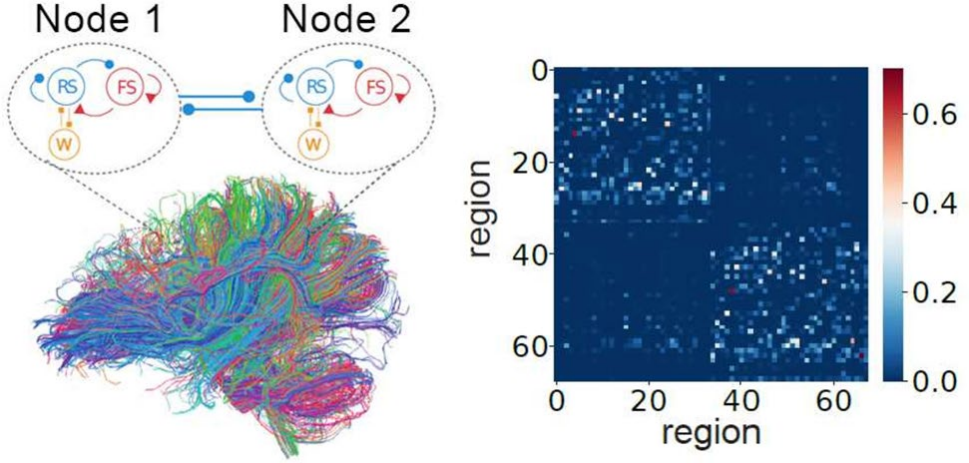
Left panel shows a diagram showing two of 68 mean-field nodes in the simulation alongside tractography data informing long-range connectivity between mean-field nodes. Right panel shows the strength connectivity matrix, $C_{j,k}$, between the 68 nodes. Reproduced with permission from (Goldman et al., 2023)

In the TVB-AdEx model, the long-range connections between mean-field models are excitatory, as is shown in the left panel of Fig. 1, and their strength is given by the structural connectivity matrix $C_{jk}$ derived from tractography data, shown in the right panel of Fig. 1.

Equation 1 describes node *k*’s evolution in time of the firing rate of the excitatory and inhibitory ($\nu _\mu ^k$ with $\mu =\{e, i\}$) populations. Equation 2 describes the covariance between populations $\lambda$ and $\eta$ ($\lambda , \eta =\{e, i\}$) in node *k* and Eq. 3 describes the evolution in time of the adaptation variable of node *k*, $W^k$. Note that Einstein’s index summation convention is used, omitting summation symbols and summing over repeated indices.

1 $$\begin{aligned} T \frac{\partial \nu _\mu ^k}{\partial t}&= (\mathscr {F}_\mu - \nu _\mu ^k) + \frac{1}{2}c_{\lambda \eta }^k\frac{\partial ^2 \mathscr {F}_\mu }{\partial \nu _\lambda \partial \nu _\eta }, \end{aligned}$$

2 $$\begin{aligned} \begin{aligned} T\frac{\partial c_{\lambda \eta }^k}{\partial t}&= \delta _{\lambda \eta } \frac{\mathscr {F}_\lambda (1/T - \mathscr {F}_\eta )}{N_\lambda } + (\mathscr {F}_\lambda - \nu _\lambda ^k)(\mathscr {F}_\eta - \nu _\eta ^k) \\ {}&\quad + \frac{\partial \mathscr {F}_\lambda }{\partial \nu _\mu } c_{\eta \mu }^k + \frac{\partial \mathscr {F}_\eta }{\partial \nu _\mu } c_{\lambda \mu }^k - 2c_{\lambda \eta }^k, \end{aligned} \end{aligned}$$

3 $$\begin{aligned} \tau _w \frac{\partial W^k}{\partial t}&= -W^k + b_e \tau _w \nu _e^k + a(\mu _V[\nu _e^k, \nu _i^k, W^k] - E_{L,e}). \end{aligned}$$

The parameter *T* sets the time scale of the mean-field models’ dynamics. Since the choice of *T* is delicate when dealing with non-stationary dynamics (di Volo et al., 2019), it has been one of the parameters included in this work’s parameter sweep. The function $\mathscr {F}_{\mu =\{e,i\}} = \mathscr {F}_{\mu =\{e,i\}} (\nu _e^{k,tot}, \nu _i^k, W^k)$ is the transfer function of the excitatory and inhibitory AdEx neurons in the spiking network, which determines the firing rate of the neuron when receiving $\nu _e^{k,tot}$ and $\nu _i^k$ as inputs. These functions are obtained through a semi-analytical derivation which is explained in detail in di Volo et al. (2019).

Parameters $b_e$ and *a* come from the AdEx single neuron model, at the microscopic scale, and stand for spike-triggered adaptation (in pA) and subthreshold adaptation (in nS), respectively. The value of *a* has been fixed to $a = 0$ nS, while different values of parameter $b_e$ will be explored during this work as we are interested in observing how the TVB-AdEx model’s activity changes when spike-triggered adaptation varies. $\mu _V(\nu _e^k, \nu _i^k, W^k)$ is a function that returns the average membrane potential of the population, obtained when deriving the mean-field model (di Volo et al., 2019). The $E_{L,e}$ parameter is also related to the single neuron model used in the spiking network. It stands for the leakage reversal potential of AdEx excitatory neurons (their resting membrane potential (Brette & Gerstner, 2005)) and its impact on the model is discussed in this work. Although it is not explicitly present in the equations, the leakage reversal potential of inhibitory neurons $E_{L,i}$ also has an important effect on the outcome of the model through both $\mu _V$ and the transfer functions, so it has also been included in the parameter sweep.

Finally, $\nu _e^{k, tot}$ corresponds to the total incoming excitatory input to a neuronal population given by:

4 $$\begin{aligned} \nu _e^{k, tot} = \nu _{aff} + \nu _{drive}^k + \sum _j SC_{jk}\nu _e^j(t - ||j-k||/v_c) \end{aligned}$$

where $\nu _{aff}$ is an afferent transient input, $\nu _{drive}^k$ is an external noise simulated by an Orsntein-Uhlenbeck (OU) process, $C_{jk}$ is the connectivity between regions *j* and *k* (with $C_{kk} = 1$) and $\nu _e^j(t - ||j-k||/v_c)$ is the activity of the excitatory population in node *j* with a delay corresponding to the distance between the regions divided by the axonal propagation speed $v_c$. The *S* parameter is phenomenologically tuned to modify the overall inter-region connectivity strength and, therefore, it is the final parameter included in this work’s analyses.

The set of differential equations are integrated using the stochastic Heun integrator from the TVB toolbox (Tvb integrators, 2023; Kloeden & Platen, 1992) where $\nu _{drive}(t) = \nu _{drive} + \sigma \xi (t)$ is the stochastic variable, where $\xi (t)$ is an OU process evolving through the following equation $d\xi (t) = -\xi (t)\frac{dt}{\tau _{OU}} + dW_t$ where $dW_t$ is a Wiener process of amplitude 1 and zero average. An integration time step of $dt=0.1 ms$ has been chosen, see an extended discussion in Section B.3

### Parameter Exploration

As previously mentioned, such a complex model contains many parameters that need to be understood and to have reasonable, physiological ranges determined for them. Most of those variables can be set based on biological or mathematical arguments (Zerlaut et al., 2017; Carlu et al., 2020), but there remains a subset of parameters whose impact needs to be studied to have a deeper and general understanding of the model. In Table 1, one can find the characteristics of the parameters chosen and the reason for their choice.

**Table 1:** Name, description, reason of choice, range and units of the parameters chosen for the parameter sweep

**Parameter**	**Description**	**Reason of choice**	**Range**	**Units**
*S*	Coupling strength between nodes	Has to be chosen phenomenologically	[0, 0.5]	Dimensionless
$E_{L, i}$	Leakage reversal potential of AdEx inhibitory neurons	Resting membrane potential of a neuron might vary depending on external conditions	$[-80, -60]$	mV
$E_{L, e}$	Leakage reversal potential of AdEx excitatory neurons	Resting membrane potential of a neuron might vary depending on external conditions	$[-80, -60]$	mV
*T*	Timescale of the AdEx mean field model	Has to be chosen phenomenologically	[5, 40]	ms
$b_e$	Adaptation strength of excitatory AdEx neurons	Models the change in neuromodulation that induces transition between AI and UD	[0, 120]	pA

For each parameter, 16 evenly spaced values are obtained inside the described range. A simulation would have been run for each of the possible combinations of parameter values, which would result in having to analyse $16^5$ differently parametrized TVB-AdEx configurations. However, preliminary results showed that neuronal activity remains silent when $E_{L, i}$ is significantly greater than $E_{L,e}$, a result of the inhibitory populations being more active than the excitatory ones. For this reason, only those combinations where $E_{L, i} < E_{L, e} + 4$ mV have been simulated. In the end, a total number of 675,840 different configurations have been analyzed.

### High Performance Computing

Though more computationally tractable than simulating a brain-scale network of spiking neurons, simulating the TVB-AdEx consisting of mean-field populations remains a relatively computationally expensive process, taking approximately a minute to simulate one second of activity on a personal computer. For each parameter combination that is going to be studied, at least five seconds of activity need to be simulated. The analyses of the simulation might take up to an extra minute to be executed. Fortunately, the simulations needed to perform a parameter sweep can be easily parallelized.

For these reasons, the use of HPC has made this parameter sweep possible. We ran our scripts on the JUSUF supercomputer in the Jülich Supercomputing Centre (Jusuf supercomputer, 2022), which consists of 187 nodes, with each node having two AMD EPYC 7742 @2.25 GHz processors for a total of 128 cores, 256GB DDR4 of RAM and 1TB NVMe for memory (Jusuf supercomputer configuration, 2022).

A profiling of a five-seconds TVB-AdEx simulation, together with the corresponding feature extraction pipeline, showed that running the script required less than 600 MB of RAM at any point in time, less than one GB of static memory for I/O operations, and approximately 6 min of execution time (see Fig. 8 in the Supplementary Information). Thus, it was possible to run 128 simultaneous simulations on each JUSUF node, increasing significantly the simulation and analysis speed.

### Feature Extraction on Spontaneous Activity

In order to visualize the results of the parameter exploration, a feature extraction pipeline has been applied, obtaining a series of metrics (or features) that represent the overall behavior of the TVB-AdEx model for each parameter combination studied (see Fig. 9 in the Supplementary Information for a schematic representation).

For each parameter combination, a five seconds simulation of the TVB-AdEx model has been run with a time step of 0.1 ms. In other words, the differential equations describing the TVB-AdEx network are numerically integrated over $5\times 10^4$ time steps.

The first two seconds following initialization of the simulation are considered a transient state and, therefore, ignored. In Sections B.1 and B.2, the interested reader can find the reasoning behind the simulation length choices. Afterwards, the array of data containing the evolution in time of the $\nu _e^ k(t)$ of the 68 regions of the model is recovered to apply the feature extraction.

In order to analyze the spectral characteristics of a parameter combination, the Fourier Transform is applied to each of the 68 time series in the $\nu _e^k(t)$ array, obtaining a $\nu _e^k(f )$ array in the Fourier space. Then, each element of the array is squared and an average over the nodes, obtaining a single *PSD*(*f*) curve representing the average Power Spectral Density (PSD) of the nodes in the network, from which spectral features will be obtained.

**Table 2:** Table containing the features that will be analyzed in this work, along with a short description of their derivation

**Feature Name**	**Feature Derivation**
**Max** $\varvec{\nu _e}$	The maximum value of $\nu _e$ for all nodes and time bins is stored.
**Mean** $\varvec{\nu _e}$	Firing rate is averaged over time and nodes of the network.
**Mean of SD of** $\varvec{\nu _e}$	The Standard Deviation (SD) of $\nu _e^k (t)$ is obtained for each node *k* and then averaged.
**Mean duration of Up states**	For each node, an algorithm detects the Up states where $\nu _e$ crosses a threshold. The mean duration of the Up states in each node is obtained. Then it is averaged over nodes.
**Frequency at peak of PSD**	A peak detection algorithm is applied to the PSD, which returns the most relevant peak in the PSD. The frequency at which this peak appears is stored.
**Mean ** $\varvec{FC}$	The Functional Connectivity (*FC*) matrix is obtained by computing the Pearson correlation between the $\nu _e$ of every pair of nodes in the network, resulting in a (68, 68) array. The mean value of this array is stored as a feature.
$\varvec{corrFCSC}$	The FC matrix can also be compared with the Structural Connectivity (SC) matrix (the connectome, $C_{j,k}$). This can be done by obtaining the Pearson correlation coefficient between the FC and SC flattened arrays.

Table 2 displays the features that will be analyzed in this report, along with a short description of how they are obtained. Other features that have been computed but whose analyses are too extensive for the limited space of this report are displayed in Table 3 in the Supplementary Information.

As a final note, the excitatory firing rate has been is the source of the features shown in this manuscript, since there is little observable difference between features obtained with $\nu _e$ and $\nu _i$.

## Results

### Normal and Paroxysmal States

First of all, it is fundamental to have a precise characterization of when the dynamics of the TVB-AdEx model exhibit firing rates in the physiological range or if they exhibit paroxysmal or aberrant activity. In this case, one, or multiple, mean-field model reaches the pathologically high activity fixed point. Since this pathological fixed point is found at around 190 Hz, well above the “normal” working range, it is possible to analyze the occurrence of this event by studying the maximum $\nu _e$ reached for every one of the 675,840 different configurations: if the maximum value of $\nu _e$ exceeds 175 Hz (activity only reaches such high values in the paroxysmal fixed point), that parameter configuration is counted as exhibiting paroxysmal dynamics.

The parameters that mostly favor the emergence of paroxysmal dynamics are *S*, $E_{L, i}$ and $E_{L,e}$ (see Fig. 15 in the Supplementary Information), which are all related to the level of excitatory/inhibitory balance in the network, consistent with previous results (Dehghani et al., 2016).

**Fig. 2:**
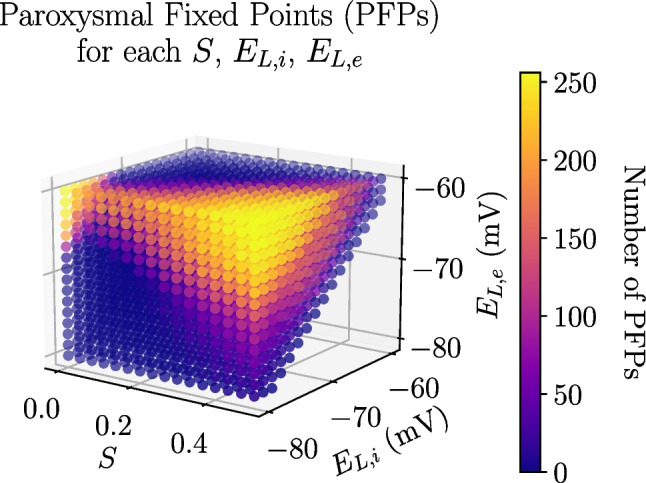
Number of Paroxysmal Fixed Points (PFPs) as a function of *S*, $E_{L, i}$ and $E_{L,e}$ parameter values

It is thus possible to build a 3D histogram, with the objective of seeing how these three different parameters interact between them to favor pathological activity, shown in Fig. 2. This is done by fixing the values of *S*, $E_{L, i}$ and $E_{L, e}$ and counting how many Paroxysmal Fixed Points (PFPs) appear when varying the remaining parameters (*T* and $b_e$).

As one could expect, the probability of reaching a PFP is largest in the region of highest *S* and $E_{L, e}$ and lowest $E_{L,i}$, since it groups together all the conditions that favor the appearance of PFPs. Curiously, one can also see that the $S=0$, high $E_{L,e}$, low $E_{L,i}$ corner also exhibits higher probability of exhibiting paroxysmal activity, indicating that disconnected mean-field modes tend to reach this pathological activity more easily. It seems that near the $E_{L,i} = E_{L,e}$ diagonal is a relatively safe working region, which we will use in the following section.

### Robustness of AI to UD Transition When Increasing Adaptation Strength

**Fig. 3:**
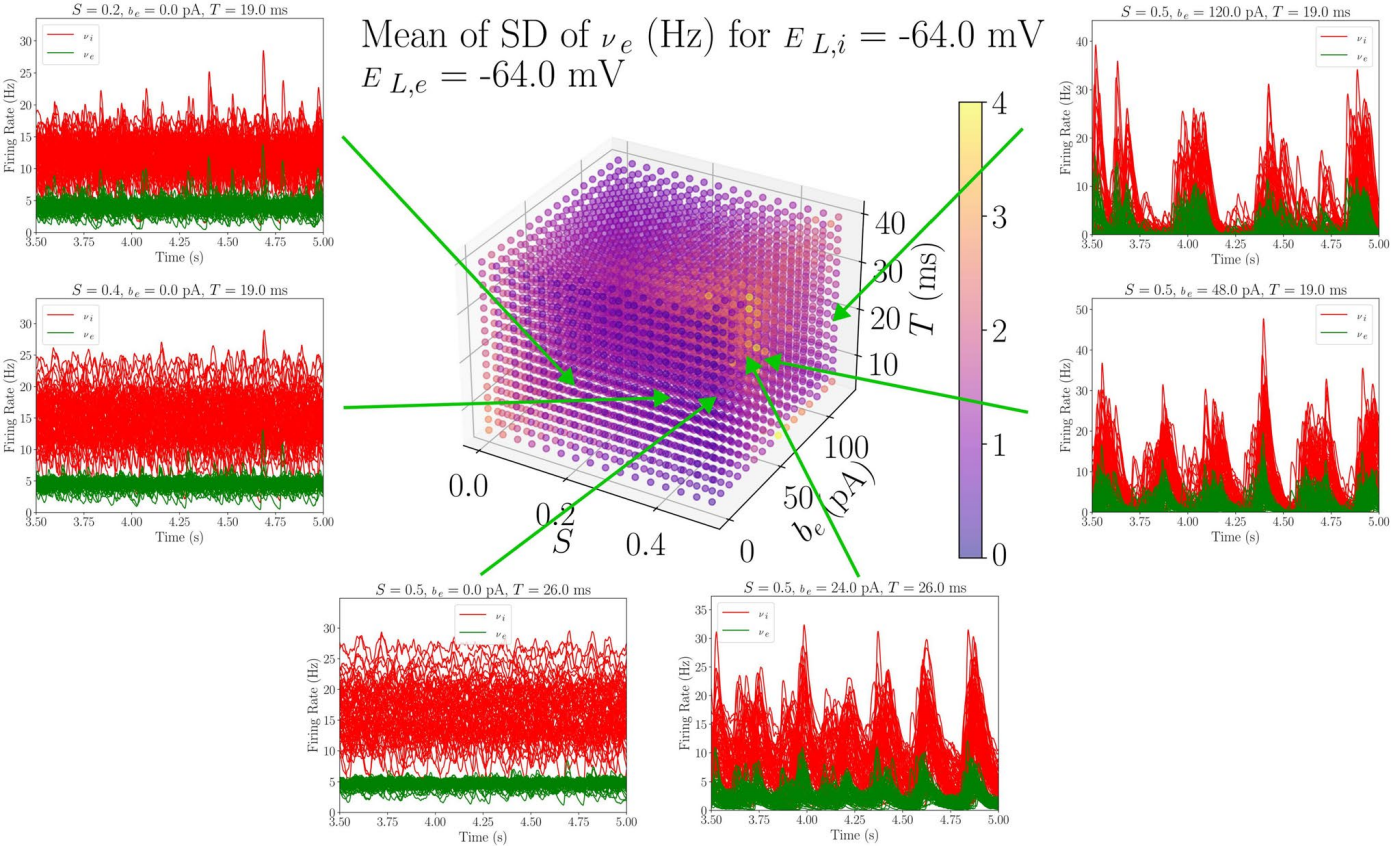
Average standard deviation values from a subspace of the *depolarized region* of the parameter space. In the floating panels, the time evolution curves of the inhibitory and excitatory populations of the 68 AdEx mean-field models are plotted for six different parameter combinations in the depolarized region ($E_{L,i} = E_{L,e} = -64$ mV). In $b_e$ = 0 pA, for intermediate values of *S*, one can see how AI states appear, consistent with the low value of SD shown in the feature plot. For $S=0.5$, one can see the transition between AI and UD states when increasing $b_e$

**Fig. 4:**
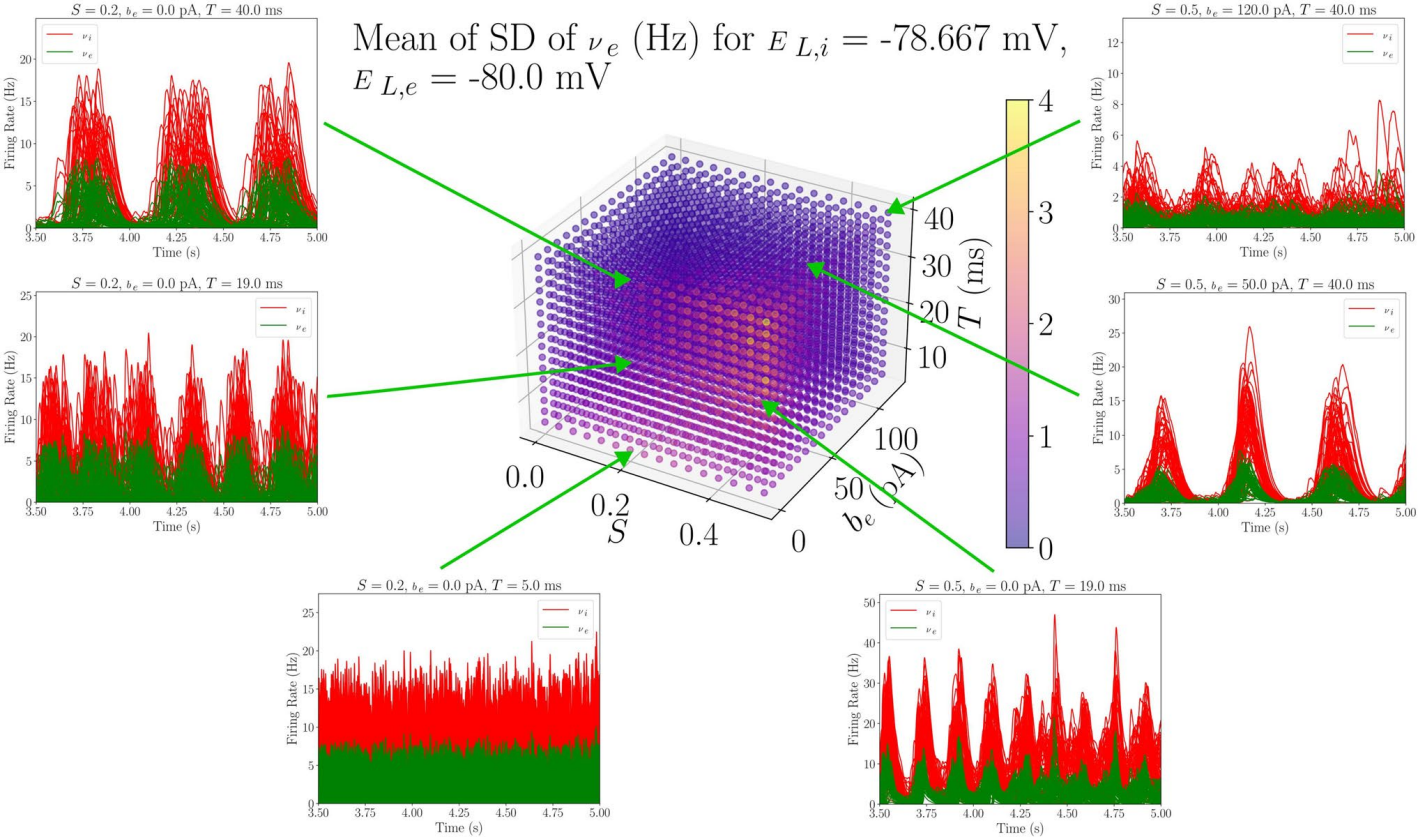
Average standard deviation values in a subspace of the *hyperpolarized region* of the parameter space. In the floating panels, the results of running the TVB-AdEx model for six other parameter combinations are plotted. In the hyperpolarized region ($E_{L,i} = -78.667$ mV, $E_{L,e} = -80$ mV) one can clearly see the effects of leakage reversal potential values on the behavior of the model. Here, AI states do not appear even for $b_e = 0$ pA and high values of *S*. Instead, this sub-region of the parameter space is dominated by UD dynamics. For small *T* values, one can see how the dynamics are very fast and desynchronized, making model behavior hard to visualize. However, since the the excitatory firing rate values reach zero, one can conclude that they consist of desynchronized UD states

The parameter sweep described in this work was designed to initially study the robustness of transitions between AI and UD states when changing the spike-frequency adaptation strength, modeled by the $b_e$ parameter. The main objective was to determine the regions of the parameter space where this transition takes place, as well as to look for other possible dynamics of the system that might relate to empirical findings. Therefore, a representative feature of the transition between AI and UD states (the $\nu _e$ standard deviation) is shown in Figs. 3 and 4 for two different regions of the parameter space, together with the time evolution of the TVB-AdEx network’s excitatory and inhibitory firing rates.

In the TVB-AdEx, AI states are described by small amplitude stochastic perturbations around a fixed point of sustained activity while UD states are described by an oscillatory traveling between the origin (Down state) and this AI fixed point (Up state). Thus, AI states are characterized by higher mean $\nu _e$, since there are no silent periods; smaller standard deviations, due to their range being narrower; and much longer Up state duration than UD states, since they are at a constant Up state.

Figure 3 shows the standard deviation (averaged over time and nodes of the network) of $\nu _e$ when fixing $E_{L,i}$ and $E_{L,e}$ at equal and relatively high values (-64 mV). We will refer to the region of the parameter space where $-60$ mV $\ge E_{L,e} \ge$ $-65$ mV as the *depolarized region*. Additionally, the time evolution of the excitatory (green) and inhibitory (red) firing rates are shown for different parameter combinations of the model (each panel corresponding to a simulation computed with the parameters of a point in the cube).

One can clearly see that, for low $b_e$ and for most *S* and *T* values, AI-like dynamics are found (three left-most panels). These patterns of activity are characterized by high mean $\nu _e$, low standard deviation, high duration of Up states and higher frequencies of *PSD* peak, which are plotted in Fig. 16 in the Supplementary Information. When increasing the spike-frequency adaptation, one can see a sharp change in the behavior (three right-most panels), towards an UD-like behavior where the standard deviation of the firing rates increases, while the mean firing rate decreases, together with the Up-state duration and the frequency at the peak of the *PSD* (again, shown in Fig. 16).

**Fig. 5:**
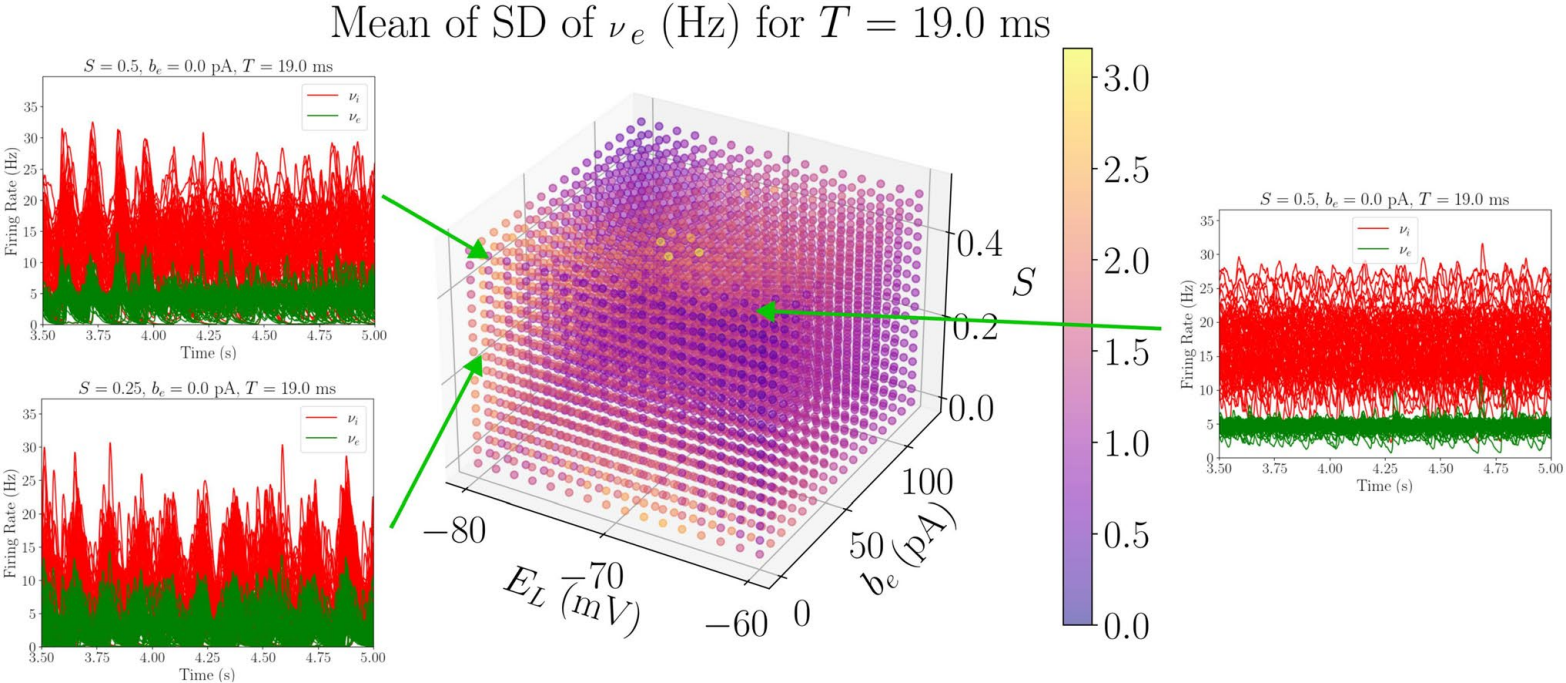
Average standard deviation values with fixed $T=19ms$, exploring $E_{L,i} = E_{L,e} = E_L$, $b_e$ and *S*. When $b=0pA$, varying $E_L$ produces a clear transition between AI (higher values of $E_L$) and UD states (low values of $E_L$), which could be associated to a descent from consciousness towards anesthetized states (Alkire et al., 2008)

It is also interesting to explore the influence of these same three parameters but in a much different region. In Fig. 4, similar plots to those in Fig. 3 are shown, but in this case, the parameters $E_{L, i}$ and $E_{L,e}$ are fixed to $E_{L,i} = -78.667$ mV and $E_{L,e} = -80$ mV, while *S*, $b_e$ and *T* are swept in the same range. In this situation, the inhibitory and excitatory populations are at the lower limit of the physiologically relevant leakage reversal potentials values (inhibitory populations are slightly less hyperpolarized for plotting purposes, to avoid some of the parosysms that may appear when $E_{L,i} = -80$ mV). Thus, we will refer to the region where $-75$ mV $\ge E_{L,e} \ge$ $-80$ mV as the *hyperpolarized region*.

Now, the standard deviation values show a non-expected peculiar behavior: the nodes of the TVB-AdEx network exhibit the highest standard deviation of $\nu _e$ for the lowest values of $b_e$, which should, in theory, correspond to AI states, associated to low standard deviation. Looking at the time evolution curves of the selected parameter combinations, it is clear that these standard deviation values come from the appearance of UD states, even when there is no adaptation in the system. This type of behavior could be associated to anesthetized brains, a state of unconsciousness where neurons are typically hyperpolarized (Alkire et al., 2008).

This modification of the behavior when $E_{L,i}$ and $E_{L,e}$ shift between the *depolarized* and the *hyperpolarized* states is relatively smooth when increasing the leakage reversal potential of the populations (increasing $E_{L,i}=E_{L,e}=E_L$ from $-80$ to $-60$ mV), as can be seen in Fig. 5. When there is no spike frequency adaptation, if the neuronal populations are hyperpolarized, UD states dominate the dynamics. However, when the neurons reach their typical $E_{L,e}$ and $E_{L,i}$ values, AI states start to appear.

Thus, this parameter sweep has shown a remarkable robustness of the transition between AI and UD states when varying the adaptation strength, $b_e$, especially in the *depolarized region* of the parameter space. Additionally, a transition from AI behavior to slow-wave oscillations has been found when hyperpolarizing the neural populations in the model, which could be related to transitions between conscious and anesthetized states.

### Relationship Between FC and SC

The parameters used in the original TVB-AdEx model in Goldman et al. (2020, 2023) show a transition from asynchronous AI to synchronized UD states, consistent with LFP empirical recordings (Destexhe et al., 1999). In order to extend that line of work, one of the objectives of this project was to investigate whether transitions from low to high synchronization between network nodes with increasing adaptation is maintained throughout the studied parameter space. Additionally, empirical studies show that the Structural Connectivity (SC), described here by the matrix $C_{j,k}$, tends to be the substrate upon which *FC* correlations appear during unconscious states, while the repertoire of *FC* patterns varies considerably and diverges from the SC in conscious states (Barttfeld et al., 2015; Tagliazucchi et al., 2015; Hahn et al., 2021). So, the relationship between the FC patterns and the SC of the TVB-AdEx model has been studied when modeling a descent towards modeled unconscious dynamics by increasing $b_e$.

To do that, the evolution of the mean value of the FC matrix, (from now on *FC*) and the Pearson correlation between the FC and SC matrix (from now on *corrFCSC*), described in Table 2, are studied as a function of $b_e$ for each combination of *S*, $E_{L,i}$, $E_{L,e}$ and *T* values. In other words, parameters *S*, $E_{L,i}$, $E_{L,e}$ and *T* are fixed and one observes how *FC* and *corrFCSC* vary when increasing $b_e$ from 0 to 120 pA, obtaining the $FC(b_e)$ and $corrFCSC(b_e)$ traces for that parameter combination. This results in 42,240 different $FC(b_e)$ and $corrFCSC(b_e)$ traces that need to be analyzed.

**Fig. 6:**
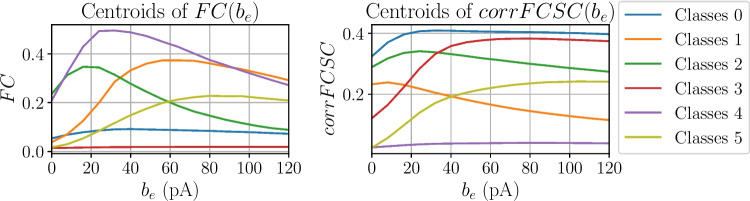
Centroids of $FC(b_e)$ and $corrFCSC(b_e)$ classes, determined by a K-means algorithm with six clusters. Note that colors are used to distinguish between classes in the same category. $FC(b_e)$ and $corrFCSC(b_e)$ classes are not related, a priori, although they share the same colors. The number of classes has been chosen for plotting purposes

To approach this cumbersome task, the $FC(b_e)$ and $corrFCSC(b_e)$ traces have been clustered (using a K-means clustering algorithm) with the objective of automatically grouping them in six $FC(b_e)$ classes and six $corrFCSC(b_e)$ classes. Those *S*, $E_{L,i}$, $E_{L,e}$ and *T* combinations containing paroxysmal activity at any point of $b_e$ were ignored from this analysis. In Fig. 6, one can see the centroids of the six $FC(b_e)$ and $corrFCSC(b_e)$ classes. In K-means clustering, the centroid of a class represents the average behavior of all the traces associated to that class, giving a general idea of their tendency.

**Fig. 7:**
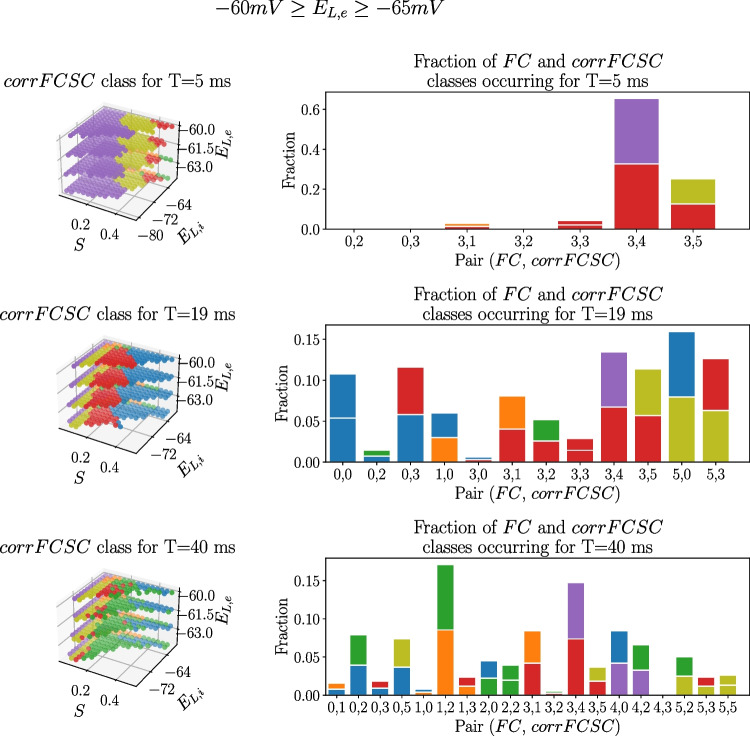
Distribution of $corrFCSC(b_e)$ classes as a function of *S*, $E_{L,i}$ and $E_{L,e}$ values. For different *T* values (left panels) and histograms represent the fraction of time a point in the parameter space is assigned to $FC(b_e)$ class *i* and $corrFCSC(b_e)$ class *j* (right panels) for the *depolarized* range of $E_{L,e}$ ($-60$ mV $\ge E_{L,e} \ge -65$ mV). The bottom color of the histogram bars are the same as the pair’s $FC(b_e)$ color, while the top color is the same as the pair’s $corrFCSC(b_e)$ color

The left panels in Fig. 7 shows the $corrFCSC(b_e)$ class for each parameter combination found in the *depolarized region* of the parameter space, while the right panels display the fraction of time that one of the parameter combinations in the left panel is assigned both $FC(b_e)$ class *i* and $corrFCSC(b_e)$ class *j*.

One can see that a low value of $T=5ms$ results in a predominance of little to no increase of *corrFCSC* with $b_e$, being purple the most frequent class (recall $corrFCSC(b_e)$ centroids from Fig. 6). Also, looking at the histogram, one can see that most $corrFCSC(b_e)$ classes are paired with the red $FC(b_e)$ class, implying that *FC* remains extremely low throughout the whole $b_e$ range for $T=5$ ms. For $T=19$ ms, both blue, red and olive $corrFCSC(b_e)$ classes appear quite frequently. Analysing the centroids in Fig. 6, blue, red and olive $corrFCSC(b_e)$ classes are the ones that show an increase in correlation when increasing $b_e$, consistent with empirical recordings (Barttfeld et al., 2015; Tagliazucchi et al., 2015; Hahn et al., 2021). Examining the histograms, one can see that the blue, red and olive $corrFCSC(b_e)$ are also paired to an interesting variety of $FC(b_e)$ classes. Finally, for $T=40$ ms, most of the points in this parameter region are assigned to the green $corrFCSC(b_e)$ class, which is characterized by a peak and slight decrease of *corrFCSC* when increasing adaptation. Although this region is the most heterogeneous of the three in terms of different class pairs, the most significant $corrFCSC(b_e)$ classes (blue, red and olive) are scarce.

The behavior of the *hyperpolarized region* is considerably different (see Fig. 18 in the Supplementary Information). In the *hyperpolarized region*, for $T=19$ ms, blue and green $corrFCSC(b_e)$ points, whose centroids start at a considerably high level of *corrFCSC*, dominate the region. This is consistent with the fact that, in the *hyperpolarized region*, one can find UD states associated to unconscious dynamics even for low values of $b_e$.

Thus, these results show that during slow-wave activity, the FC patterns of the TVB-AdEx model typically become more constrained by the anatomical connectivity than during conscious-like states, which is in close agreement with the empirical results obtained in Barttfeld et al. (2015), Tagliazucchi et al. (2015), and Hahn et al. (2021), especially for intermediate values of *T*, such as $T=19$ ms.

## Discussion and Future Perspectives

In this work, using HPC resources, we have explored the large-scale activity of the TVB-AdEx whole brain model throughout a large parameter space spanned by microscopic parameters. We found that transitions between conscious and unconscious-like dynamics when increasing adaptation strength $b_e$ are largely robust, especially when the leakage reversal potential of excitatory populations is $-60$ mV $\ge E_{L,e} \ge$ $-65$ mV. Moreover, we showed that it is possible to obtain slow-wave activity, similar to unconscious brain states, when strongly hyperpolarizing the neural populations of the model via $E_{L,i}$ and $E_{L,e}$, independently of the level of spike-frequency adaptation. Finally, we found that the FC patterns arising from unconscious-like dynamics are usually closer to the underlying anatomical connectivity than those from conscious-like states, which is consistent with results from several empirical studies (Barttfeld et al., 2015; Tagliazucchi et al., 2015; Hahn et al., 2021).

Studying Paroxysmal Fixed Point probability in the TVB-AdEx model, one finds that the total number of PFPs in the parameter space is considerable, where approximately one fifth of the tested parameter combinations reaches this pathological fixed point. The parameters that most strongly modulate the probability of reaching a PFP are *S*, $E_{L,e}$ and $E_{L,i}$, which are able to modify the balance between excitation and inhibition in the neural populations. This high number of PFPs could be decreased by applying a stronger long-range excitatory coupling on inhibitory populations with respect to excitatory populations (Li et al., 2021), to compensate for the imbalance between excitation over inhibition that causes pathological activity. This asymmetry could be further investigated to relate values of $E_{L,e}$ and $E_{L,i}$ to experimental findings of resting membrane potentials in different areas of the cortex. Because these paroxysmal points exhibit excessively high firing rates, and appear generally when there is an excess of excitation, they resemble epileptic-type activity. Although the TVB-AdEx produces epileptic activity, the resolution will be improved in the future with further biophysical detail to include the direct representation of other microscopic mechanisms underlying epilepsy, for instance, K^+^ dynamics (Depannemaecker et al., 2021).

The timescale of the model, expressed through parameter *T*, has a powerful influence on the dynamics of the TVB-AdEx model. Apart from strongly modulating the frequency of oscillations of neuronal activity (clearly seen in Figs. 16 and 17), it affects the overall level of synchronization and how the FC patterns relate to the anatomical connectivity matrix (Figs. 7 and 18).

In the TVB-AdEx network, low values of *T*, such as $T=5$ ms, can result in violating one of the necessary assumptions used to build the AdEx mean-field model: that the system is Markovian over a certain time scale *T* (El Boustani & Destexhe, 2009). This violation results in aberrant, noisy and fast oscillating activity (an example of this has been shown in Fig. 4 bottom-left panel). Therefore, correlations between nodes become extremely rare, resulting in low levels of mean FC and *corrFCSC* independently of the adaptation strength value. For large values of the timescale parameter, such as $T=40$ ms, the dynamics of the model slow down considerably, and correlations between regions increase, especially in UD states. This increase homogenizes the FC matrix, which leads to a greater dissimilarity between the FC and SC matrices, thus explaining the overall decrease in *corrFCSC* as a function of $b_e$ for $T=40$ ms. Additionally, for large values of *T*, the system remains memoryless. However, inputs arriving to networked mean-field models at frequencies faster than 1/*T* (around 25 Hz for $T=40$ ms) appear as a constant external drive (di Volo et al., 2019). Therefore, the results obtained for intermediate values of *T*, such as $T=19$ms, support the idea that it is necessary to use a slow enough timescale to not violate the Markovian assumption, but fast enough to keep the loss of high-frequency information to a minimum.

Although the TVB has been shown to effectively mimic both conscious and unconsciouss-like large-scale dynamics throughout the studied parameter sweep, the model still has several noteworthy limitations.

First of all, this version of the TVB-AdEx model uses an anatomical substrate representing purely cortical regions and their connectivity and therefore does not directly represent subcortical dynamics (Alkire et al., 2008; Aru et al., 2019). Rather, the TVB-AdEx model relies on simplified representations of incoming subcortical effects of transient and noisy inputs, modeled as $\nu _{aff}$ and $\nu ^k_{drive}$, respectively, from Eq. 4. Therefore, future advances should include cortical-subcortical loops, particularly, the explicit representation of thalamo-cortical connections in TVB-AdEX.

Additionally, this multi-scale cortical model assumes that each region shares the same parameters as the others. For instance, one could try to make use of an acetylcholine receptor density map to obtain a heterogeneous distribution of $b_e$ parameters, similarly to what is done in Herzog et al. (2023). Future iterations of the TVB-AdEx model will therefore need to run even more quickly to efficiently scan parameter space.

Finally, most current empirical functional connectivity studies use fMRI data (Barttfeld et al., 2015; Tagliazucchi et al., 2015; Hahn et al., 2021) to record brain activity at long temporal scales, with a typical sampling rate of two seconds, while the sampling time of the TVB-AdEx model is of 0.1 ms. While we report progress in biophysically bridging between spatial scales in neural simulations, bridging between time scales is out of the scope of this manuscript. Bridging time scales will be explored in later work, profiting from the TVB library fMRI simulator (BOLD monitor), which is resource intensive, requiring long simulations to compare with empirical fMRI data. In the TVB-AdEx model, there is typically an increase of the mean FC value with increased adaptation, as has been shown in Section 3.3. However, this is not the case in fMRI signals, where the mean FC value decreases during the descent to deep sleep (Hahn et al., 2021). An intriguing possibility is that this discrepancy might be related to differences in time scales, which should be further investigated. However, even with HPC, the parameter space is too large to assess efficiently. Thus, reducing the computational cost of long TVB-AdEx model simulations would be extremely beneficial, as it would allow the use of TVB’s BOLD monitor to simulate fMRI signals. Analyzing simulated BOLD signals would allow to better relate the model’s behavior to empirical fMRI findings. Also, the reduction of computational cost would allow to repeat a similar parameter sweep but studying the network properties of the FC matrix, in order to determine whether the TVB-AdEx model can also simulate known fMRI FC characteristics of brain pathologies (Bullmore & Sporns, 2009; Fornito et al., 2015; Farahani et al., 2019). Therefore, we propose RateML as tool that would allow to speed up simulations by allowing the user to generate accelerated CPU and GPU-ready TVB models (van der Vlag et al., 2022).

In recent years, multiscale brain modeling has gained prominence (Breakspear, 2017; D’Angelo & Jirsa, 2022), ultimately leading to the development of virtual brain models (D’Angelo & Jirsa, 2022; Leon et al., 2013). These virtual whole brain models have been extensively researched and applied to address diverse neurological issues, including epilepsy (Jirsa et al., 2017; Olmi et al., 2019), neurodegenerative diseases (Alexandersen et al., 2023; van Nifterick et al., 2022), and the exploration of various states of consciousness (Goldman et al., 2020, 2023; Herzog et al., 2023; Cakan et al., 2022).

Despite the recent progress in whole brain modeling, to the best of our knowledge, only the models presented by Cakan et al. (2022) and the TVB-AdEx, presented by Goldman et al. (2020, 2023), have demonstrated successful simulations of slow oscillations, associated to deep sleep and unconscious states. Furthermore, in Goldman et al. (2020, 2023), the authors show that Perturbational Complexity Index (PCI) of the TVB-AdEx model decreases when the spike frequency adaptation parameter, $b_e$, is increased, simulating the reported decrease of PCI when losing consciousness (Casali et al., 2013). Therefore, we have further analyzed the behavior of the TVB-AdEx model by conducting an extensive exploration of the parameter space, which serves to provide a more comprehensive characterization of the model’s behavior. Additionally, we have also analyzed the relationship between functional and structural connectivity within the model, a characteristic closely tied to consciousness (Barttfeld et al., 2015; Tagliazucchi et al., 2015; Hahn et al., 2021).

In Hahn et al. (2021), Hahn et al. constructed a whole brain model that successfully simulated the functional connectivity (FC) and the similarity between FC and structural connectivity (SC) for humans and primates, both falling asleep and under different levels of anesthesia. However, the model used in Hahn et al. (2021) simulated the BOLD signals of each brain area with the normal-form of the Hopf bifurcation, which lacks biological inspiration and interpretation. In this study, we have shown that the TVB-AdEx is a biologically-inspired multiscale model that also simulates an increase in similarity between FC and SC during the descent into deep sleep, coinciding with the appearance of slow-wave activity. However, in Hahn et al. (2021), both the empirical recordings and the presented model showcase a decrease in the mean value of the FC when slow-wave dynamics emerge, while the TVB-AdEx tends to show an increase in FC. As previously mentioned, this divergence could potentially be attributed to the timescales being simulated and will be further explored in future studies.

Together, these results show that the TVB-AdEx model is a flexible and robust multi-scale model, where changes of microscopic parameters lead to transitions in the dynamical behavior of simulated activity, where the emergent activity states display properties in general agreement with experimental measurements.

### Supplementary Information

Below is the link to the electronic supplementary material.

Supplementary file1 (PDF 10.7 MB)

Supplementary file2 (ZIP 7.97 KB)

## Data Availability

The code used for the simulations presented in this paper can be found in https://github.com/davidaquilue/TVBAdEx_ParSweep and will be available online and in EBRAINS after publication. All the data produced on the HPC simulations and used to perform the analyses can be found inside the code repository: https://github.com/davidaquilue/TVBAdEx_ParSweep/tree/main/JUSUFlike/Project/Data.
