# Supplementary material for: High-Density Exploration of Activity States in a Multi-Area Brain Model
Source: Neuroinformatics. 2023 Nov 20;22(1):75–87. doi: 10.1007/s12021-023-09647-1 (PMC10917847; doi:10.1007/s12021-023-09647-1)
Supplement: Supplementary file 1 — Supplementary file1 (PDF 10.7 MB) [file 12021_2023_9647_MOESM1_ESM.pdf]

High-Density Exploration of Activity States in a Multi-Area Brain Model
Supplementary Information

David Aquilué-Llorens, Jennifer S. Goldman, Alain Destexhe

Abstract

To simulate whole brain dynamics with only a few equations, biophysical, mesoscopic models of local neuron populations can be connected using empirical tractography data. The development of mesoscopic mean-field models of neural populations, in particular, the Adaptive Exponential (AdEx mean-field model), has successfully summarized neuron-scale phenomena leading to the emergence of global brain dynamics associated with conscious (asynchronous and rapid dynamics) and unconscious (synchronized slow-waves, with Up-and-Down state dynamics) brain states, based on biophysical mechanisms operating at cellular scales (e.g. neuromodulatory regulation of spike-frequency adaptation during sleep-wake cycles or anesthetics). Using the Virtual Brain (TVB) environment to connect mean-field AdEx models, we have previously simulated the general properties of brain states, playing on spike-frequency adaptation, but have not yet performed detailed analyses of other parameters possibly also regulating transitions in brain-scale dynamics between different brain states. We performed a dense grid parameter exploration of the TVB-AdEx model, making use of High Performance Computing. We report a remarkable robustness of the effect of adaptation to induce synchronized slow-wave activity. Moreover, the occurrence of slow waves is often paralleled with a closer relation between functional and structural connectivity. We find that hyperpolarization can also generate unconscious-like synchronized Up and Down states, which may be a mechanism underlying the action of anesthetics. We conclude that the TVB-AdEx model reveals large-scale properties identified experimentally in sleep and anesthesia.

A Supplementary Methods

A.1 Profiling for HPC computing

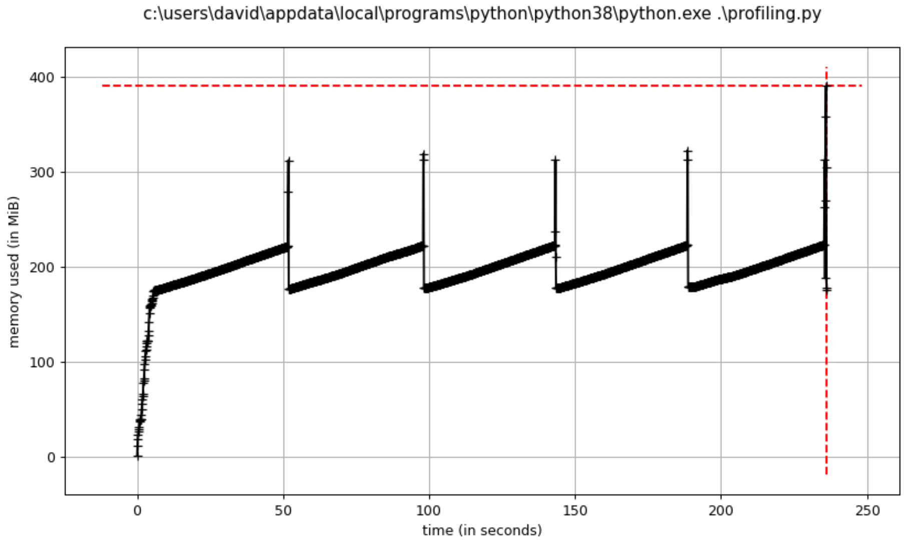

Figure 8: Memory and time consumption profiling for a single TVB-AdEx model simulation, without feature extraction. One can see that the amount of Random Access Memory (RAM) needed at each point never exceeds 400 MB. The feature extraction pipeline makes sure to only use the necessary resources, never surpassing the 600 MB limit. Thus, it is possible to exploit the 128 cores of a JUSUF node without worrying about exceeding the 256 GB RAM limit.

A.2 Detailed Description of Features

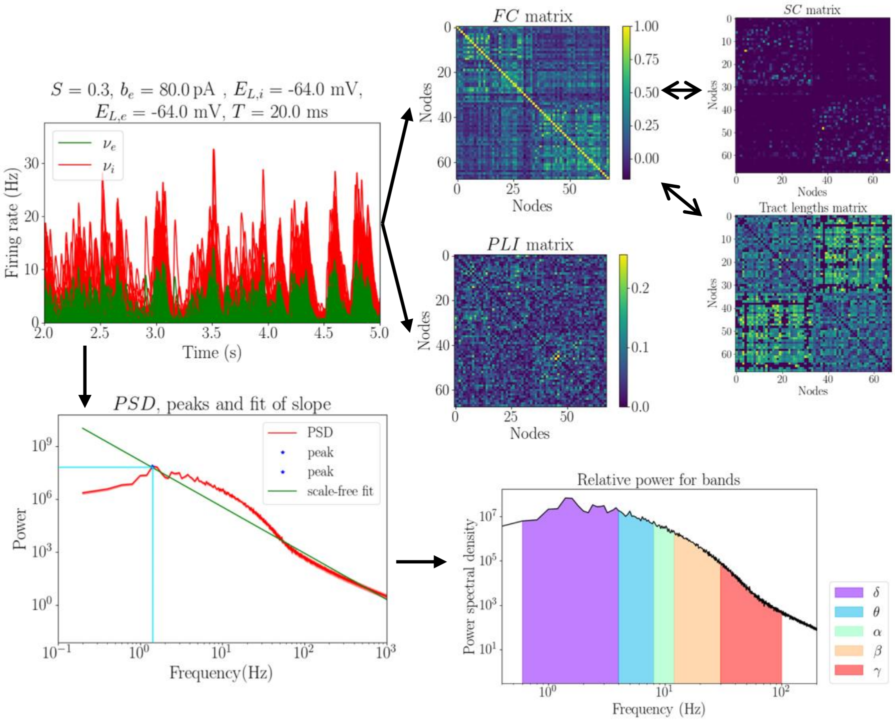

Figure 9: Feature Extraction pipeline. For each parameter combination, the features described in Tables 1 and 3 are obtained. All the features stem from the raw time-traces of the model, from which the FC matrix, the PLI matrix and the $PSD(f)$ are obtained. The FC matrix is compared with both the SC matrix and the Tract Lengths matrix, and also used for a study on Default Mode Network correlations, explained below. Then, different algorithms are applied on the $PSD(f)$ to obtain the spectral features.

Feature Name	Feature Derivation
**Mean duration of Down states**	For each node, an algorithm detects the Up states where ν_e crosses a threshold. A Down state is the time between the moment when ν_e becomes smaller than the threshold and crosses it again. The mean duration of Down states in each node is obtained. Then it is averaged over nodes.
**Mean PLI value**	The average Phase Locking Index (PLI) of the whole network is obtained by averaging the PLI matrix, result of computing the PLI value for each pair of nodes in the network. See Appendix of [10].
**Power Law of PSD**	The PSD of neuronal activity is expected to follow a Power Law of the type $PSD(f) = \frac{b}{f^a}$. In a loglog plot, this is equivalent to fitting a linear regression, from which we store the a value.
**Score of PSD Power Law**	The r-score obtained when fitting the linear regression on the loglog plot of the PSD is stored.
**Relative Powers of frequency bands**	5 bands are defined in the (0.5, 100) Hz range of frequency: $\delta \in$: (0.5, 4) Hz, $\theta \in$ (4, 8) Hz, $\alpha \in$ (8, 12) Hz, $\beta \in$: (12, 30) Hz, and $\gamma \in$: (30, 100) Hz. The $\int_{B_i}^{B_f} PSD(f)df$ gives the spectral power in a band. Dividing by the total power (integrated between 0.5 and 100 Hz) we obtain the fraction of spectral power in a band.
corrFCTL	Similar to the $corrFCSC$, the Pearson correlation between the flattened Functional Connectivity Tract Lengths (TL) matrices is obtained. The TL matrix indicates the length of the anatomical tracts of white matter between two regions in the whole brain parcellation.
**Ratio top z-score of DMN**	The Default Mode Network (DMN) are a set of regions in the brain that are correlated during resting brain dynamics (when the brain is not performing any cognitive task). It has also been shown that its elements remain correlated when falling asleep. It is also believed that it is strongly tied to anatomical pathways [25, 26, 27].<br>For these reasons, since one of the strengths of the model is its anatomically informed connectivity, correlations between regions associated to the DMN were studied. The regions considered as the DMN for the given parcellation can be seen in the Figure immediately below. As done in fMRI studies, one of the regions (left precuneus) is taken as a seed and the correlations between the seed and all the other regions are obtained (Fisher transformation is applied to obtain a zscore). Then, the resulting metric is obtained by calculating how many of the DMN components appear in the top ten most correlated anatomical regions with the seed. One would expect that most of the DMN components appear in this top ten, however, results have not been conclusive.

Table 3: Remaining features that have been obtained for each parameter combination but that have not been included in the main text's analyses. It is expected to make use of them in further work.

B Supplementary results

B.1 Long-term Meta-Stability of Up and Down States

In this section, we show the time evolution curves for the excitatory and inhibitory firing rates of the mean field models in the TVB-AdEx network during the last four seconds of a 100 seconds long simulation, for two different parameter combinations that exhibit UD states. These two plots show that the UD states described throughout this work and the work of Goldman and colleagues [1, 2] truly are a meta-stable state of the TVB-AdEx model.

With increased time of simulation, the dynamics do not converge to a single fixed point, but instead each mean-field model exhibits the meta-stable UD states.

Thus, we argue that simulating no more than five seconds is already sufficient to characterize the dynamics of the model, as well as necessary to minimize the already large computational cost of the parameter space exploration.

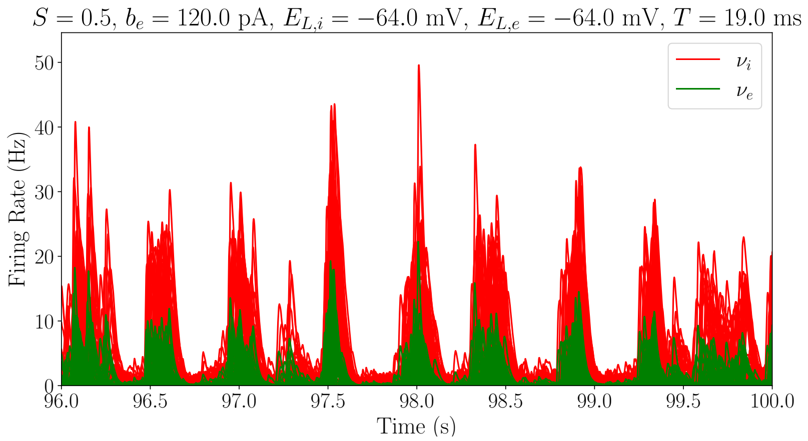

Figure 10: 100 seconds simulation for a parameter combination in the *depolarized* region of the parameter space with high b_e, exhibiting sustained UD states even in long simulations.

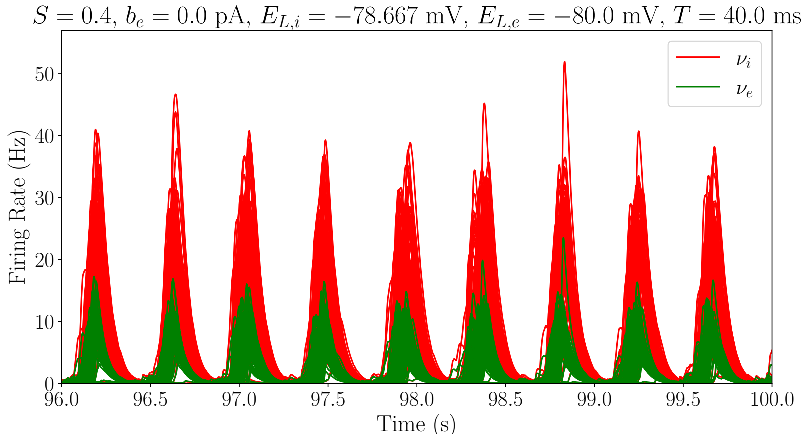

Figure 11: 100 seconds simulation for a parameter combination in the *hyperpolarized* region of the parameter space with low spike frequency adaptation parameter b_e, exhibiting sustained UD states even in long simulations.

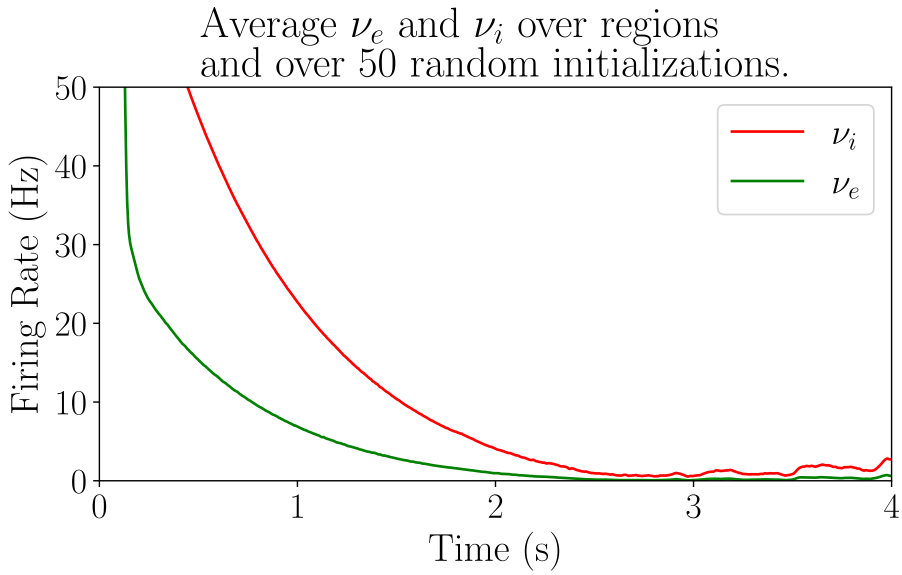

Figure 12: Average and Standard deviation of the excitatory and inhibitory firing rates for multiple initializations. Statistics aggregated both over different initializations and over all the mean-field models in the network.

B.2 Duration of transient state after initialization

When numerically integrating a system of differential equations, one usually ignores a portion of the integrated trajectories after initialization. This is done so that possible artifacts that might arise from starting the integration at a point far away from the stable dynamics of the model. These meaningless dynamics are called *transient states*, and are typically not relevant for posterior analyses of the behavior of the model.

Therefore, it is important to choose an adequate length of time to remove from the analyses. Ideally, one would choose to remove an exceptionally large amount of integrated time steps to ensure that the analyzed dynamics are not contaminated by transient states.

However, the computational cost of running the five-dimensional parameter exploration described in this work restricts the length of time the simulation can be integrated. Thus, one must find a trade-off between avoiding transient states, reducing computational cost, and obtaining long enough trajectories for adequate analyses.

Figure 12 shows how the initialization of the simulation impacts the model's behavior for the parameter combination $S = 0.5$, $b_e = 120$ pA, $E_{L,i} = -64$ mV, $E_{L,e} = -64$ mV, $T = 19$ ms. For this parameter combination, $M = 50$ four-second-long simulations have been generated, each simulation m with different initial $\nu^k_{e,0,m}$, $\nu^k_{i,0,m}$ values, with $\nu^k_{e,0}, \nu^k_{i,0} \in [0, 30]$ Hz. For each simulation m, all the mean-field models in the network, described by index k, are initialized with the same $\nu^k_{e,0,m}$, $\nu^k_{i,0,m}$ values.

In Figure 12, the averaged $\langle \nu_e \rangle_{m,k}(t)$ and $\langle \nu_i \rangle_{m,k}(t)$ are shown, where $\langle \cdot \rangle_{m,k}$ is an average over the $M = 50$ different simulations and the $K = 68$ regions

in the TVB-AdEx network. The shaded area shows the standard deviation, computed following the same rationale.

These two metrics help visualize the typical duration of the transient state. For this particular combination, it is interesting to note that the transient state reaches its end shortly after $t = 2$ s, when the the statistics change from a smooth to a noisy curve, a result of the appearance of UD states. Additionally, at $t = 2$ s, the ranges of values the statistics of the firing rates take resemble the values of the statistics well after the transient state, near the end of the simulation.

The parameter combination shown here has been specifically chosen as it is highly likely to have a particularly long transient state. On the one hand, as shown in Figure 15, the high connectivity strength S increases the probability of reaching a paroxysmal fixed point (PSP) at the beginning of the simulation. On the other hand, the high value of spike frequency adaptation will rapidly increase whenever the PSP is reached, which will then drive the dynamics back to their *healthy*, working, state, requiring of a long transient state to go from the PSP to the typical UD regime. Thus, by having studied the length of this transient state, we argue that removing the first two seconds of a simulation provides a good trade off between avoiding possible transient states while allowing us to keep the computational cost of the parameter exploration manageable.

B.3 Effect of Numerical Integration Time Step

In this section we analyze the impact that a smaller time step of integration has on a wide range of features that have been previously described in Table 1 in the manuscript and Table 3 in this Supplementary Information. By varying the adaptation parameter b_e when performing this analysis, it is possible to visualize the trends the set of features exhibit when increasing spike frequency adaptation.

A sweep of b_e from 0 to $80pA$ has been performed, in steps of $20pA$. For each b_e value, a 5 second-long simulation has been computed and analyzed, following the same pipeline as in the main text, for two different values of dt: $dt = 0.1$, which has been used throughout this study, and $dt = 0.01$, a shorter integration time step.

In Figure 13, temporal, functional, and spectral features of the TVB-AdEx are shown for the two previously described time steps, as a function of b_e. The results clearly indicate that, for the vast majority of the features analyzed, the results are highly consistent, independently of the integration time step used, thus justifying the use of a larger time step of $dt = 0.1$ for reduced computational time and resources. Nonetheless, functional features exhibit clear differences at certain spike frequency adaptation values, specifically the mean value of FC. But, although it seems that the mean value of FC is significantly different, specially at the $b_e = 40pA$ mark, one can infer from the $corrFCSC$ point that the FC matrices have similar structure, as they follow the same trajectory of similarity with the SC matrix. Additionally, smaller integration time steps result in having more samples, which can help capture correlations.

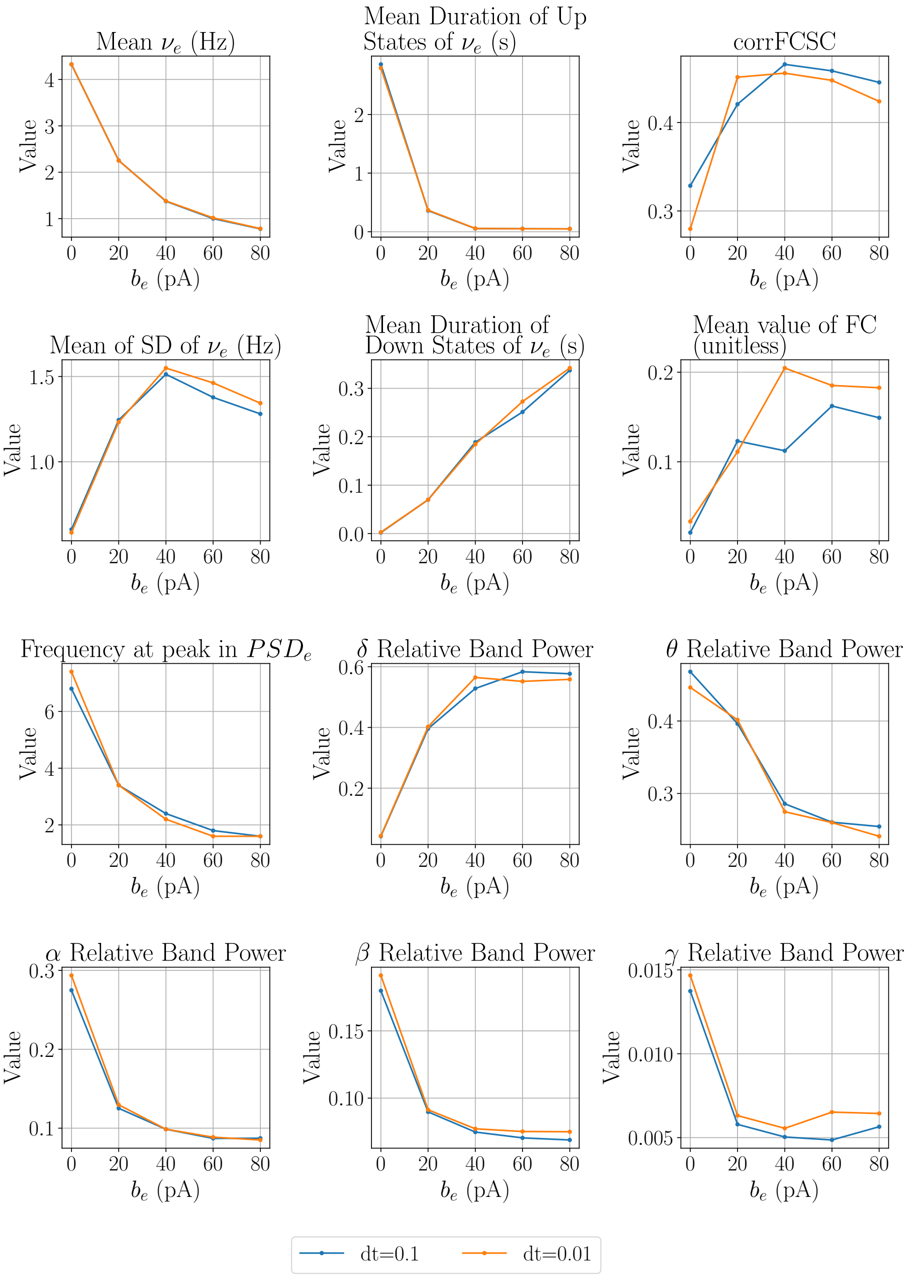

Figure 13: Comparison of the impact of different integration time step sizes for an extensive set of features. These features have been used to analyze the behavior of the TVB-AdEx model throughout this work and describe temporal (Mean and Mean of SD of ν_e, Mean Duration of Up and Down states), functional ($corrFCSC$ and Mean value of FC), and spectral (frequency at peak of PSD and relative band powers) characteristics.

B.4 Impact of parameter values on paroxysmal activity

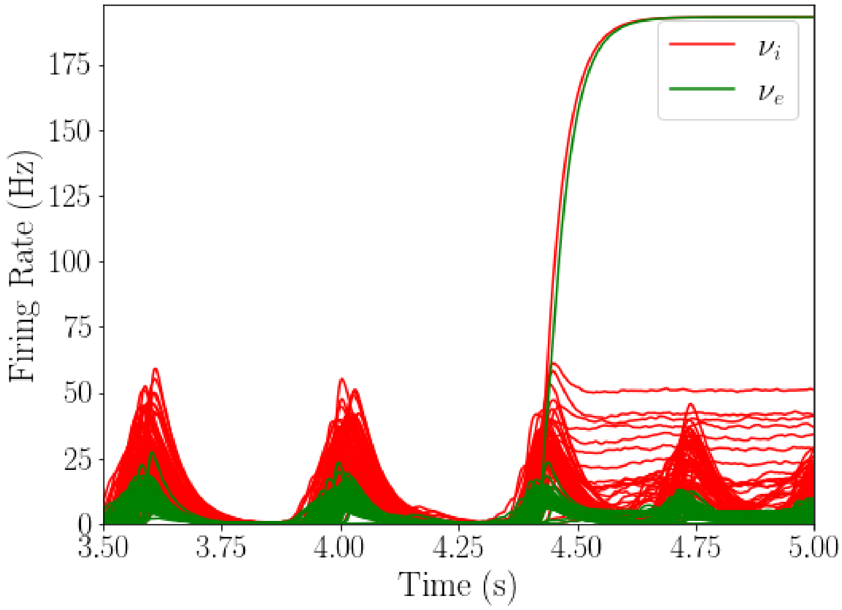

Figure 14: Example of how the ν_e of one region in the TVB-AdEx model jumps to the pathological activity fixed point around 4.5 s.

To analyze the influence of each parameter on the appearance of Paroxysmal Fixed Points (PFPs) of the TVB-AdEx model, one can count, for all the parameter combinations containing a certain fixed parameter value, how many times the ν_e threshold of 175 Hz is exceeded. That way, representations like the one in 15 can be built.

The excitatory transfer function of the AdEx mean field model ($\mathscr{F}_e$ from Equations 1 and 2 in the manuscript is such that when it receives a high ν_e input, the attractor can change, making the dynamics jump to the pathological fixed point at around 192 Hz. Thus, higher ν_e incoming to the AdEx mean field models in the network will make it easier to reach this pathological activity. It is worth noting that a single AdEx mean-field model is an already complicated model to analyze dynamically. Propositions made in this appendix should be thoroughly tested through dynamical analyses in further work to better understand the mean-field model behavior.

Increased values of connectivity strength between nodes, S, result in higher ν_e firing rates incoming to all the mean-field models in the network, considerably increasing the probability of reaching a PFP. Both leakage reversal potential, $E_{L,i}$ and $E_{L,e}$, play a similar but inverse role. When $E_{L,i}$ takes lower values, the inhibitory populations of the network's nodes become hyperpolarized, less active, leading to a higher excitatory activity, increasing the probability of going over the threshold for PFPs. When $E_{L,e}$ takes lower values, the excitatory populations are less active, and the chances of reaching a PFP are lower, but when the leakage reversal potential increases, making excitatory populations more active, the probability rapidly increases. It is interesting to note how

the b_e value has almost no influence on PFPs, since the probability of a PFP appearing remains almost constant for the whole b_e range. Finally, one can see in Figure 15 that PFP probability increases steadily with T. Increasing T makes the dynamical system slower in such a way that the trajectory of the ν_e towards the fixed point is slower after a perturbation. Thus, incoming perturbations that move the trajectory towards higher activity can accumulate, each time increasing more and more the ν_e, until the transition line is crossed and the AdEx mean-field model is attracted by the 192 Hz fixed point.

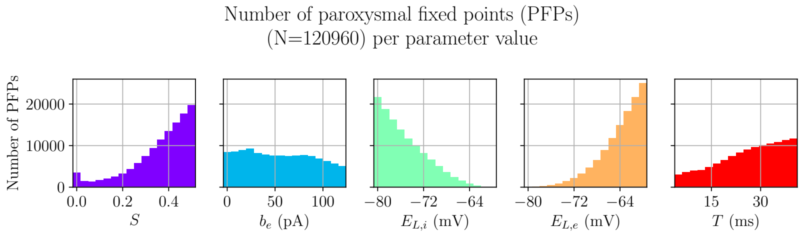

Figure 15: Histograms of number of times a Paroxysmal Fixed Point happens as a function of parameter values.

B.5 Additional features demonstrating the behaviour of the TVB-AdEx Model in the depolarized and hyperpolarized regions of the parameter space.

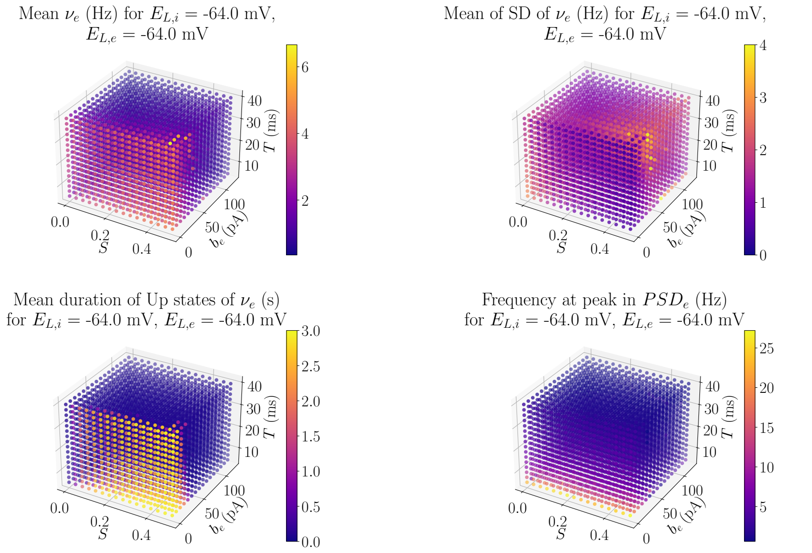

Figure 16: Features describing the behavior of the TVB-AdEx model in a subspace of the *depolarized region*, when varying S, b_e and T.

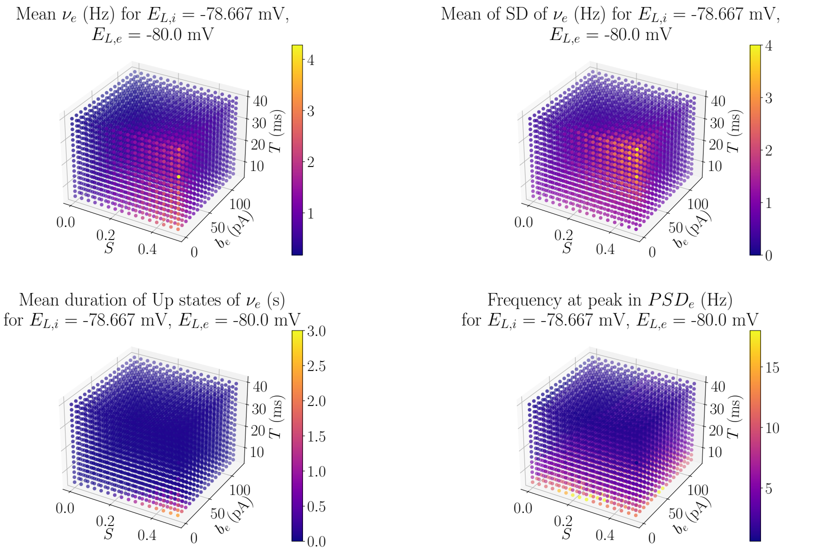

Figure 17: Features describing the behavior of the TVB-AdEx model in the *hyperpolarized region*, when varying S, b_e and T.

B.6 Relationship between FC and SC. Hyperpolarized Region

$$-75mV \geq E_{L,e} \geq -80mV$$

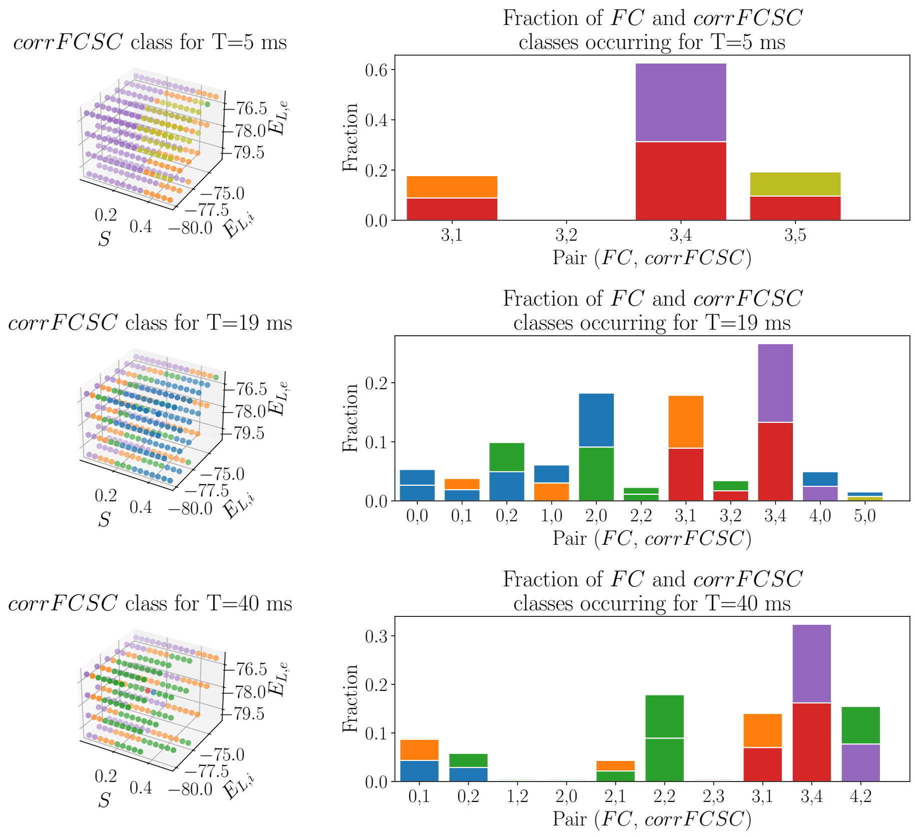

Figure 18: Distribution of $corrFCSC(b_e)$ classes as a function of S, $E_{L,i}$ and $E_{L,e}$ values for different T values (left panels) and histograms counting the fraction of times a point in the parameter space is assigned to $FC(b_e)$ class i and $corrFCSC(b_e)$ class j (right panels) for the *hyperpolarized* range of $E_{L,e}$ (-75 mV $\geq E_{L,e} \geq -80$ mV). The bottom color of the histograms' bars are the same as the pair's $FC(b_e)$ color, while the top color is the same as the pair's $corrFCSC(b_e)$ color.

The behavior of the *hyperpolarized region* is considerably different to that of the *depolarized region*. In this case, it is hard to see homogeneous regions and the diversity of $FC(b_e)$, $corrFCSC(b_e)$ pairs is reduced. Again, for $T = 5$ ms, the only $FC(b_e)$ class available is the red one, indicating a total lack of overall connectivity in the TVB-AdEx network. In the $T = 19$ ms plots there is an interesting appearance of many blue and green $corrFCSC(b_e)$ points, which are related to those centroids that start at a considerably high level of $corrFCSC$. This is consistent with the fact that, in the *hyperpolarized region*, one can find UD states associated to unconscious dynamics even for low values of b_e. Lastly,

for $T = 40$ ms, the green $corrFCSC(b_e)$ class becomes the most frequent one again, as was the case of Figure 7, now being mostly paired with purple and green $FC(b_e)$ classes, which correspond to very high levels of FC throughout the b_e range.

References

[1] Jennifer S. Goldman, Lionel Kusch, Bahar Hazal Yalçinkaya, Damien Depannemaecker, Trang-Anh E. Nghiem, Viktor Jirsa, and Alain Destexhe. Brain-scale emergence of slow-wave synchrony and highly responsive asynchronous states based on biologically realistic population models simulated in the virtual brain. *bioRxiv*, 2020.

[2] Jennifer S. Goldman, Lionel Kusch, David Aquilué Llorens, Bahar Hazal Yalçinkaya, Damien Depannemaecker, Kevin Ancourt, Trang-Anh E. Nghiem, Viktor Jirsa, and Alain Destexhe. A comprehensive neural simulation of slow-wave sleep and highly responsive wakefulness dynamics. *Frontiers in Computational Neuroscience*, 16:1058957, 2023.
